# Effect of time windows in LSTM networks for EEG-based BCIs

**DOI:** 10.1007/s11571-022-09832-z

**Published:** 2022-07-01

**Authors:** K. Martín-Chinea, J. Ortega, J. F. Gómez-González, E. Pereda, J. Toledo, L. Acosta

**Affiliations:** 1 Department of Industrial Engineering, University of La Laguna, 38071 San Cristóbal de La Laguna, Tenerife Spain; 2 Department of Computer and Systems Engineering, University of La Laguna, 38071 San Cristóbal de La Laguna, Tenerife Spain

**Keywords:** EEG, LSTM, Brain–computer interface, Machine learning, Deep learning, Artificial neural network

## Abstract

People with impaired motor function could be helped by an effective brain–computer interface (BCI) based on a real-time electroencephalogram (EEG) and artificial intelligence algorithms. However, current methodologies for interpreting patient instructions from an EEG are not accurate enough to be completely safe in a real-world situation , where a poor decision would place their physical integrity at risk, such as when traveling in an electric wheelchair in a city. For various reasons, such as the low signal-to-noise ratio of portable EEGs or the effects of signal contamination (disturbances due to user movement, temporal variation of the features of EEG signals, etc.), a long short-term memory network (LSTM) (a type of recurrent neural network) that is able to learn data flow patterns from EEG signals could improve the classification of the actions taken by the user. In this paper, the effectiveness of using an LSTM with a low-cost wireless EEG device in real time is tested, and the time window that maximizes its classification accuracy is studied. The goal is to be able to implement it in the BCI of a smart wheelchair with a simple coded command protocol, such as opening or closing the eyes, which could be executed by patients with reduced mobility. Results show a higher resolution of the LSTM with an accuracy range between 77.61 and 92.14% compared to traditional classifiers (59.71%), and an optimal time window of around 7 s for the task done by users in this work. In addition, tests in real-life contexts show that a trade-off between accuracy and response times is necessary to ensure detection.

## Introduction

New technological developments increasingly require non-physical interaction with the user. These types of applications not only cover routine or leisure aspects, but facilitate the lives of people who may have impaired motor function, (e.g. patients with amyotrophic lateral sclerosis (ALS) (Miao et al. 2020), spinal injuries, head trauma, rehabilitation, etc.) (Pfurtscheller et al. 1998; Craik et al. 2019). In recent years, many works have studied the development of brain–computer interfaces based on a non-invasive EEG device, from the standpoint of the design of both paradigms and algorithms for extracting features from EEG signals (Miao et al. 2020). In applications where the BCI is used by patients with some pathology, a BCI with maximum precision is essential for user safety. The difficulty with this application is that the classifier must make a decision in most cases based on a single trial (it is unable to obtain an averaged trial for the feature extraction). There are currently many research works aimed at finding better tools to identify an instruction and make a decision (Taniguchi et al. 2007; Li et al. 2016). A wide variety of methods for acquiring brain activity focus on non-invasive devices, such as magnetoencephalography (MEG), positron emission tomography (PET), and functional magnetic resonance imaging (fMRI). Even though electroencephalography (EEG) signals by nature have a poor signal–noise ratio, especially when acquired with wireless EEG devices, we conducted our research with this device due to the low cost, comfortability and ease of installation for a future user.

The first fact to consider is that the EEG signal patterns vary among users, and do not even remain constant over time for the same user. They always change in any patient state: when someone is at rest, performing some task (such as moving a hand) or is acoustically stimulated. Therefore, finding user responses to stimuli in real time, as well as determining the duration required for the signal recording to be classified properly, are essential to building an efficient BCI. All this time-dependent information and changes in the patient’s status extracted from the EEG signals have to be taken into account to design a classifier that improves decision-making in any application executed in real time. Real-time execution poses additional problems. The first one, as Netzer et al. defined in their paper, is a common issue in BCI processing, namely that the probability of success in the detection depends on capturing time (Netzer et al. 2020). If the BCI has short reaction time, the classification algorithm will perform worse, while if a better algorithm is used, the reaction time increases considerably. For example, they propose an algorithm based on coreset to calculate the common spatial patterns (CSP), keeping its representation with better results. The second problem is fatigue, as Roy et al. demonstrated. Mental fatigue can affect the signal intensity in EEG due to the distribution of these band power features, which are affected by the emotional and physical states (Roy et al. 2013). Complementarily, Myrden and Chau (2015) found a lower BCI performance caused by fatigue in their experiments involving a navigation game in which the participants had to report their level of fatigue, inter alia.

As a result of the complexity of the EEG interpretation, especially in a real-time application, the extraction and classification of information requires a complex methodology based on machine learning (ML) or deep learning (DL) techniques. Despite many existing classification algorithms in artificial intelligence-based applications, we could consider several of the most commonly applied algorithms. Lotte and others in their articles (Lotte et al. 2007, 2018) review the most frequently used classification algorithms in EEG-based applications, highlighting the following methods: discriminant analysis (DA), support vector machine (SVM), artificial neural network (ANN), k-nearest neighbor classification algorithm (KNN), decision tree learning (DTL), etc., which have yielded high accuracies, although not enough to apply these systems in an actual environment. The DA and SVM algorithms rely on a discriminant hyperplane search to identify classes, and they are prevalent in EEG-based BCI. In DA, it attempts to separate the data into different classes using a hyperplane, assuming that the data has a normal distribution and each class has an equal covariance matrix. The SVM searches for the hyperplane that maximizes the margin of the training data to the hyperplane. Algorithms based on artificial neural networks (ANN) assemble artificial neurons that are used to establish non-linear decision limits, but these are generic algorithms that could be negatively affected by noisy and non-stationary signals, such as those from an EEG. The KNN algorithm classifies new data according to the distance of the k nearest neighbors (Poorna et al. 2017). And the DTL is based on recursive partitioning of the instance space to predict the response (Wang et al. 2009; Ishfaque et al. 2013).

All these algorithms need a set of features extracted from the EEG signals as input data. However, due to the large number of features that can be obtained, the features needed for each classification method have to be pre-screened; that is, from each trace performed, a number of specific features is extracted (average power of a band in electrodes, connectivity, symmetry, etc.), losing the temporal information present in the brain when executing a task.

Against this backdrop, and given the importance of deep learning in a biomedical context (Zemouri et al. 2019), a classifier based on neural networks that can remember the information could provide a suitable alternative for an improved BCI. There is a type of recurrent neural network (RNN)—Long short-term memory (LSTM)—which is able to meet this requirement. The LSTM was developed by Hochreiter and Schmidhuber (1997). It is an RNN with the ability to remember information for long periods and forget unnecessary information, which means that the result outputs are based on a temporal pattern (Hochreiter and Schmidhuber 1997; Gers et al. 2000). It has been applied to EEG records, for example, to predict epileptic seizures (Tsiouris et al. 2018) or for emotion recognition (Alhagry et al. 2017; Li et al. 2018; Yang et al. 2018; Xing et al. 2019). Additionally, other researchers have applied this kind of RNN in similar contexts to ours: Kumar et al. (2019), who applied it in the BCI competition IV dataset 1 by combining common spatial pattern (CSP) with this type of network; Nagabushanam et al. (2020), who compare two specific neural network architectures (one with an LSTM layer) with the SVM; Li et al. (2016) defined an architecture and applied an LSTM net to classify motor imagery tasks in a public database, and something similar was applied by Wang et al. (2018) who classify motor imagery tasks of a public dataset by using their proposed framework based on LSTM, although they used a fixed time window.

The aims of this work are to study the effectiveness of using an LSTM with a low-cost wireless EEG device like Emotiv EPOC + working in real time, and to find the minimal optimal time window that maximizes the accuracy of the LSTM classifier. These objectives will lay the foundations for achieving a low-cost BCI that could be used by people with impaired motor function, for example, in a smart electric wheelchair with basic commands based on a simple code of opening and closing the user’s eyes. In order to determine the performance of using LSTM, we compared our results with popular ‘classical’ classification algorithms applied to EEG-based BCI, such as DA, SVM, KNN and DTL. In addition, the results obtained with the LSTM algorithms are compared with those obtained with a conventional recurrent neural network (RNN).

## Materials and methods

**Fig. 1:**
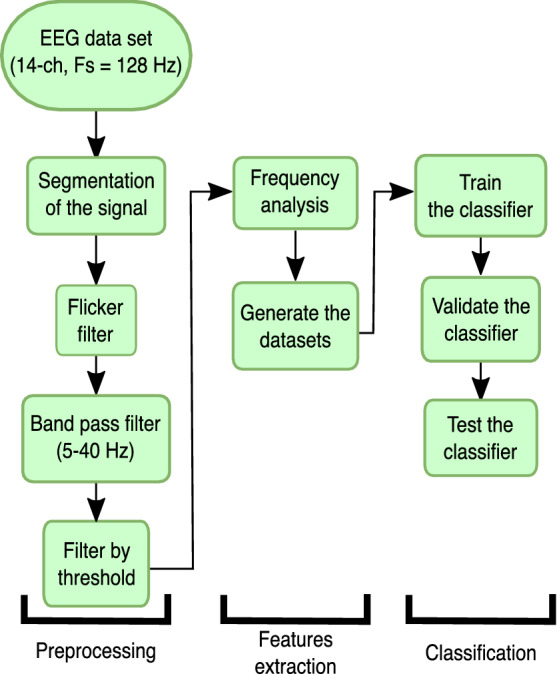
Flowchart for EEG signal pre-processing, feature extraction and classification

This section aims to define and explain the methods carried out in this research in an effort to highlight the relationship between the time window size and the robustness of a BCI, which follows the workflow shown in Fig. 1.

The flowchart for EEG signal pre-processing, feature extraction and classification is given in Fig. 1, which starts with the data acquisition using the Emotiv EPOC + to register the entire 14-channel EEG signal at a sample frequency of 128 Hz. Each data trial was divided into two seconds. A denoising of eye-blinks, a band-pass filter and a threshold were applied to remove any possible noise and artifacts. The flow continues by generating all the individual datasets with this processed signal, which consisted of different sequence sizes. The signal is then subject to a frequency analysis to obtain its power. Finally, a common train, validation and test processing is carried out to review the performance of the system.

### Data acquisition and analysis software

**Fig. 2:**
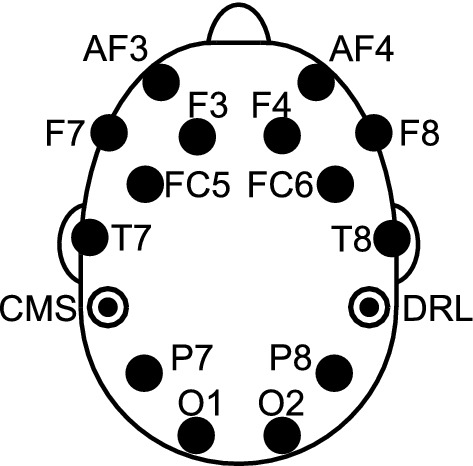
Location of the Emotiv EPOC + headset electrodes as per the 10–20 system, where CMS and DRL are references electrodes at P3 and P4, respectively

The EEG signals were recorded by the Emotiv EPOC + device in combination with the Emotiv Xavier Testbench v3.1.21 software at a sampling rate of 128 Hz. Emotiv EPOC + is a wireless, low-cost and non-invasive EEG device with 16 sensors (14 channels at positions AF3, F7, F3, FC5, T7, P7, O1, O2, P8, T8, FC6, F4, F8, AF4 and two reference electrodes at P3 (CMS, Common Mode Sense) and P4 (DRL, Driven Right Leg)), as per the 10–20 system, Fig. 2.

All the analyses in this paper were conducted using Matlab® (The MathWorks, Inc.). The data were analyzed with Fieldtrip (Oostenveld et al. 2011), a toolbox developed by the Donders Institute for Brain, Cognition and Behaviour in Nijmegen (Netherlands), in collaboration with other institutes. This toolbox offers pre-processing and advanced analysis methods for MEG, EEG, iEEG and NIRS records. In addition, the LSTM was defined, trained and tested with the Matlab Deep Learning Toolbox™. The statistical computing (Cohen 1988) was carried out using R software (R Core Team 2019).

### Participants

All the volunteers, a total of 13, gave their consent before the experiment. They ranged in age from 18 to 50 and all of them were right-handed, with no motor pathologies. The study was approved by the ethics committee of the University of La Laguna (register number: CEIBA2020-0405).

### Experimental protocol

**Fig. 3:**
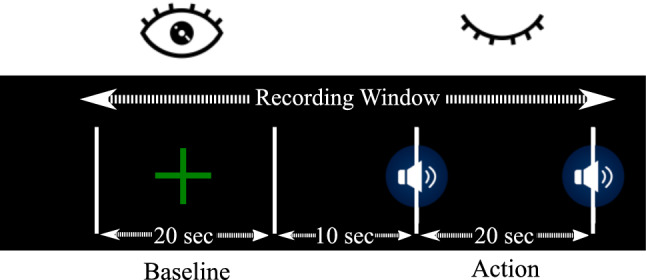
Experimental protocol. Each trial consisted of a 20-s fixation period with eyes open and a cross on the screen, 10-s rest with a black screen and 20-s period with eyes closed. A beep marks when to close or open the eyes. EEG data were recorded during the entire time

The experimental paradigm is defined in Fig. 3. This protocol was fully recorded, and it is composed of two important parts, both lasting 20 s with a 10-s rest period in between.

In the first part of the experiment, a cross was displayed, and the volunteer did not have to do anything. This is considered the baseline state, during which the rest state of the EEG is recorded. The second part started and finished with an acoustic stimulus (beep) to indicate when to close and open their eyes, respectively. These actions, each lasting 20 s, were divided into different time window sizes for classification purposes.

### Data pre-processing

The pre-processing consists of three steps: A denoising of eye-blinks, a band-pass filter and an automatic artefact rejection.

The denoising of eye-blinks was done using the REBLINCA method performed in Di Flumeri et al. (2016)**,** where the regression component of the *FPZ* channel (*Regr-FPZ*) was calculated. The reason for applying this procedure is to eliminate the muscular effects of flicker on the signal, and thereby exclusively classify the EEG signal. Because our EEG device does not have the necessary central sensor, we defined a reference signal (*AFZ*) as the average of the AF3 and AF4 channels. The regression component of this signal (*Regr-AFZ*) was calculated using a 5th-order Butterworth filter between 1 and 7 Hz. A threshold signal (*Thres-AFZ*) was computed as the derivative of the *Regr-FPZ* in order to highlight both rising and falling parts generated by the eye blink. This signal was then normalized to have a mean of zero and a variance of one. Finally, a moving average was applied over the square of the signal. The *Thres-APZ* component was used to locate where the eye blink disturbance was. Then, in the blinking areas, the original signal was corrected by subtracting the weighted *Regr-FPZ* signal for each channel:

1 $$x_{ch} (t) = x_{ch} \left( t \right) - w_{ch} *RegrAPZ\left( t \right)$$

where we previously estimated the weight of the proportion of the signal AFZ presented in each channel as:

2 $$w_{ch} = \frac{{mean\left( {abs\left( {x_{ch} \left( t \right)} \right)} \right)}}{{mean\left( {abs\left( {AFZ\left( t \right)} \right)} \right)}}$$

**Fig. 4:**
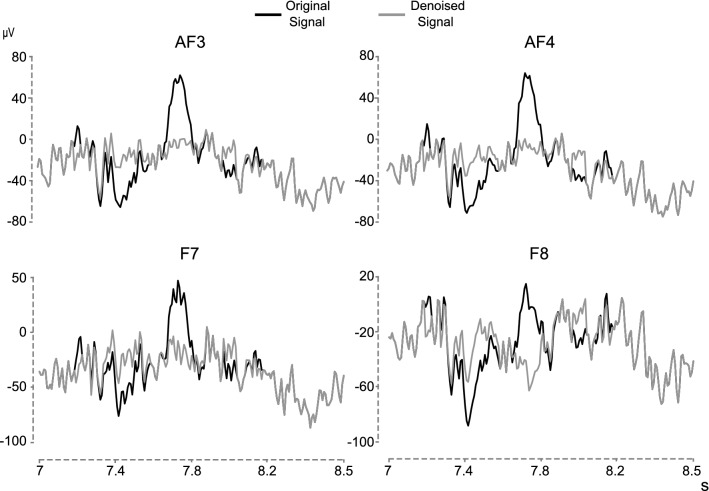
Denoising example for eye-blink. The black lines represent the EEG signals recorded at positions AF3, AF4, F7 and F8 when an eye-blink occurs. The gray lines are the same recordings without the eye-blink artefact after applying the REBLINCA method (Di Flumeri et al. 2016) adapted to the Emotiv EPOC + device

Figure 4 shows an example of an eye blink subtraction in four sensors (AF3, AF4, F7 and F8) where these artefacts are highlighted.

Then, the Butterworth band-pass filter between 5and 40 Hz was applied to the data.

Finally, a threshold (*thre*_*j*_) was computed for the automatic artefact rejection as defined in the Fieldtrip tutorial (Donders Centre for Cognitive Neuroimaging 2019): the signal of each channel (*x*_*ch*_*(t)*) is standardized (*z*_*ch*_*(t)*) with respect to its standard deviation (*σ*_*ch*_) and its average (*µ*_*ch*_) over time (*t*):

3 $$z_{ch} \left( t \right) = \frac{{x_{ch} \left( t \right) - \mu_{ch} }}{{\sigma_{ch} }}$$

where

4 $$\mu_{ch} = \frac{1}{N} \mathop \sum \limits_{t = 1}^{N} x_{ch} \left( t \right)$$

with N being the total number of samples.

After that, a new signal (*z*_*sum*_*(t)*) is created as the average of all the standardized data of each channel, where *c* is the number of channels:

5 $$z_{sum} \left( t \right) = \mathop \sum \limits_{ch = 1}^{c} z_{ch} \left( t \right)/\sqrt c$$

The threshold (*th*_*rej*_*)* to remove the outliers, which were considered artefacts and noise, is defined as:

6 $$th_{rej} = \mu_{sum} + 3\sigma_{sum}$$

where µ is the average and *σ* is the standard deviation of the signal *z*_*sum*_

7 $$\mu_{sum} = \frac{1}{N} \mathop \sum \limits_{t = 1}^{N} z_{sum} \left( t \right)$$

8 $$\sigma_{sum} = \sqrt {\frac{1}{N} \mathop \sum \limits_{t = 1}^{N} (z_{sum} \left( t \right) - \mu_{sum} )^{2} }$$

with N being the total number of samples.

**Fig. 5:**
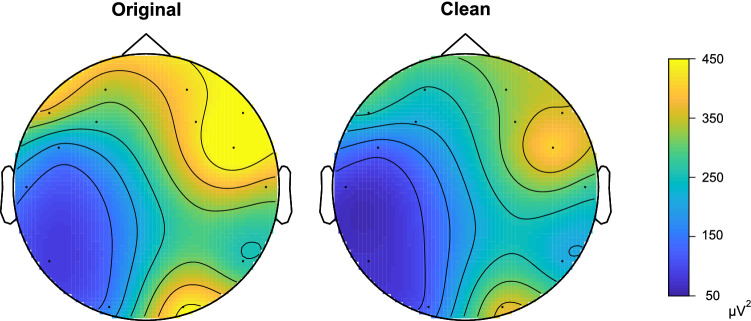
Threshold example application on a segment of closed-eyes state. The left topographic map corresponds to the power before the application of the threshold, and the right one to the power after it

An example of the application of this threshold to an EEG user test is shown in Fig. 5.

### Power and dataset generation

After the signal pre-processing, the Morlet Wavelet Transform (Tallon-Baudry and Bertrand 1999) is applied to calculate the evolution of the power in the time–frequency domain. Because the purpose of a classifier is to discern when a user is in the eyes-open (EO) or eyes-closed (EC) state, the alpha band (8–12 Hz) of the EEG signals in the visual area (occipital lobe) corresponding to O1 and O2 sensors must be analyzed (Barry et al. 2009).

Since the classical classification algorithms selected (SVM, DA, KNN, and DTL) and the LSTM network use the same features in different formats, two databases were generated. The LSTM dataset was created with time sequences of the power in O1 and O2 with a fixed time window for all trials of 20 s by adding their corresponding label (EO or EC),

9 $$\left\{ {\left( {\left\{ {P_{O1,i,j} , i = 1, \ldots ,n_{w} } \right\}, \left\{ {P_{O2,i,j} , i = 1, \ldots ,n_{w} } \right\}, E_{j} } \right), j = 1, \ldots ,n_{s} } \right\}$$

where *n*_*w*_ is the number of points in a sequence, *n*_*s*_ is the number of sequences and *E*_*j*_ is the label.

**Fig. 6:**
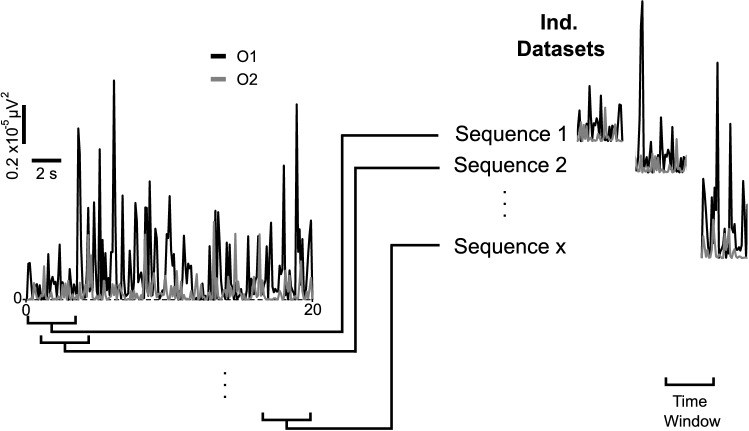
Dataset generation for LSTM network, showing an example of time-varying alpha-band power records recorded at position O1 (black trace) and O2 (gray trace) used by the LSTM algorithm. The power traces are segmented (sequences 1, 2,…, x) for use by the LSTM, by taking the power acquired in a time window, starting backwards from the point in question

The time window, which defines the duration of the sequence, was modified from 1 to 10 s to study the LSTM performance. Each sequence was created by moving the window along the signals, always back from the last sample (Fig. 6).

The dataset used in the classic algorithms was created with the power measurements from all the 20-s trials in each time in O1 and O2 adding its corresponding label (EO or EC),

10 $$\left\{ {\left( {P_{O1,i} ,P_{O2,i} ,E_{i} } \right), i = 1, \ldots ,N} \right\}$$

where *N* is the number of samples and *E*_*i*_ is the label. In the case of these algorithms, it is possible to do extra pre-processing to work with sequences in a similar way to LSTMs. But in this research, the aim is to apply the same database for all algorithms with no additional transformations and to see both the metrics obtained and the result in a real case.

## Classification

### LSTM algorithm

**Fig. 7:**
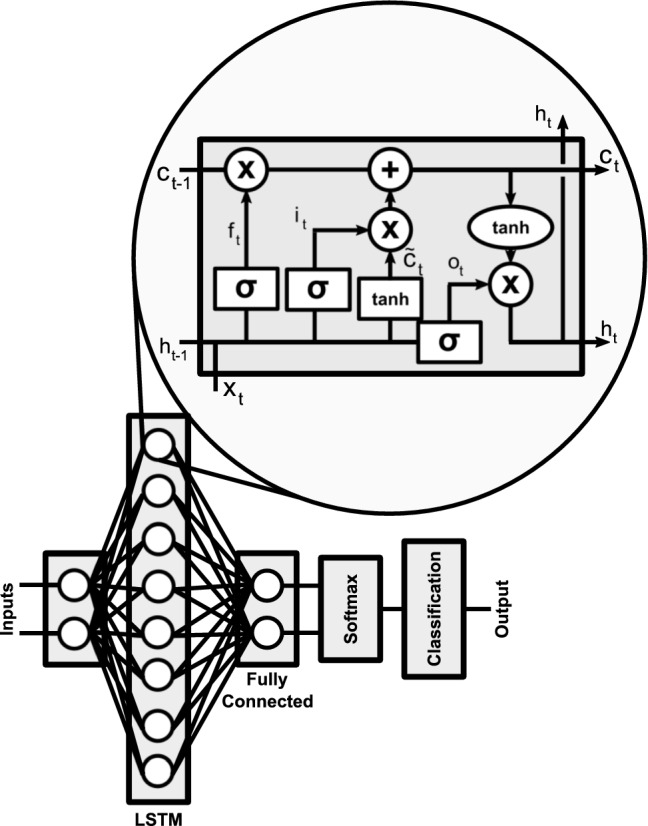
Architecture of the neural network used in this work. The LSTM layer has cells, the structure of which is shown in the exploded view, where x_t_ is the input, h_t_ the output and c_t_ is the state

The classification algorithm selected is an ANN with an LSTM layer (Hochreiter and Schmidhuber 1997). The complete neural network architecture is shown in Fig. 7. This architecture was composed of a sequence input layer (with two neurons corresponding to the features selected for each channel, O1 and O2), an LSTM layer with eight cells (this layer can learn long-term dependencies between time steps of the sequence data), a fully connected layer (which connects every neuron in one layer with the other one) and a SoftMax layer (which applies the SoftMax activation function to define the probability of each class) and, finally, the classification layer to define the outcome (Kudo et al. 1999; Bishop 2006).

The most important layer is the LSTM. The top of Fig. 7 shows the cell, where each square in the cell is a layer of the neural network that corresponds to a sigmoid or tanh operation. The circles represent specific operations like vector multiplication ‘x’, vector sum ‘+’ or a tanh operation. The arrows define the data flow, including the following operations: merging of two lines, concatenation or bifurcation. This cell uses the following data: input from the recurrent network (*x*), cell output (*h*) and memory (*c*). Each kind of data changes as a function of time *t*.

The structure of a cell can be divided into different parts. The horizontal line at the top of the diagram returns the state of the cell, and the data defined in it can be added or removed from specific gates. The first interaction in the state of the cell is generated for a sigmoid layer that decides what information is irrelevant from the *h*_*t*−*1*_ and *x*_*t*_ sources, and deletes the data on the state of the cell (*f*_*t*_).

11 $$f_{t} = \sigma \left( {W_{f} \cdot\left[ {h_{t - 1} , x_{t} } \right] + b_{f} } \right)$$

The cell input selection can be divided into two steps. In the first step, a sigmoid layer defines the key values and updates the most important ones (*i*_*t*_).

12 $$i_{t} = \sigma \left( {W_{i} \cdot\left[ {h_{t - 1} , x_{t} } \right] + b_{i} } \right)$$

In the second step, a combination of new candidate data ($\tilde{c}_{t}$) is generated by a tanh layer and past data.

13 $$\tilde{c}_{t} = tanh\left( {W_{c} \cdot\left[ {h_{t - 1} ,x_{t} } \right] + b_{c} } \right)$$

Finally, the data to forget (*f*_*t*_) are removed and the previously selected data ($\tilde{c}_{t}$) are added as described in the following formula to generate the newly updated cell status (*c*_*t*_).

14 $$c_{t} = f_{t} *c_{t - 1} + i_{t} *\tilde{c}_{t}$$

A more detailed explanation of how LSTMs work is provided by Hochreiter et al. (Hochreiter and Schmidhuber 1997).

### Classic algorithms

The LSTM algorithm was compared with the following common classification algorithms used in the literature (Lotte et al. 2007).

#### Support vector machine (SVM)

The SVM algorithm searches for a hyperplane in a space of n dimensions, where n is the number of classes, which separates the classes with a margin as wide as possible, with the margin being the width of the region parallel to the hyperplane. In this algorithm, multiple kernels that map the input data to a higher dimension space could be applied. By applying these kernels, the computational effort and time can be reduced, and the algorithm is able to deal with non-linear problems.

#### Discriminant analysis (DA)

Classification method that assumes that different classes have their data based on different Gaussian distributions. Based on this assumption, the classification is done by calculating the probability that a new element belongs to each trained class.

#### k-nearest neighbor (KNN)

Lazy learning method that classifies at the time the new data are tested. This algorithm defines the label of a new element based on the closest known labels, the closest neighbors. This requires a distance function, meaning several distance functions as kernels were used.

#### Decision tree learner (DTL)

This algorithm is based on a tree structure. This algorithm defines a root node and creates forks, or branches, according to the training parameters until it reaches a leaf node, which defines the final label. Starting from the root, the label of a new element can be defined, since at each point its values are evaluated, defining the branch to follow until the leaf node is reached.

#### Recurrent neural network (RNN)

Recurrent neural network similar to feedforward networks, but with a recurrent connection that has an associated tap delay. This allows the network to have an infinite dynamic response to time series input data.

### Parameters and kernels

Since the fit parameters and the dimensionality of the data can affect the classifiers themselves, several parameters and kernel functions have been applied (Table 1).

**Table 1:** Description of the different classical algorithm options

Algorithm	Option	Description
DTL	Fine	Creates many leaves to make fine distinctions between classes (max. number of divisions is 100)
	Medium	Uses a medium number of leaves to distinguish classes more finely (max number of divisions is 20)
	Coarse	Makes coarse distinctions between classes using few leaves (max. number of splits is 4)
DA	Linear	Differentiates classes by creating linear boundaries
	Quadratic	Differentiates classes by creating nonlinear boundaries
SVM	Linear	Uses a linear separation to distinguish classes
	Quadratic	Uses a quadratic separation to distinguish classes
	Cubic	Uses a cubic separation to distinguish classes
	Fine Gaussian	Applies a kernel scale set to sqrt(P)/4 to make very detailed distinctions between classes, where P is the number of predictors
	Medium Gaussian	Applies a kernel scale set to sqrt(P) to make medium distinctions between classes, where P is the number of predictors
	Coarse Gaussian	Applies a kernel scale set to sqrt(P)*4 to make coarse distinctions between classes, where P is the number of predictors
KNN	Fine	The number of neighbors is set to 1 to make fine distinctions between classes
	Medium	The number of neighbors is set to 10 to make medium distinctions between classes
	Coarse	The number of neighbors is set to 100 to make coarse distinctions between classes
	Cubic	Medium distinctions between classes are sought with a cubic distance metric and 10 neighbors
	Weighted	Medium distinctions between classes are sought with a weight distance metric and 10 neighbors
	Cosine	Medium distinctions between classes are sought with a cosine distance metric and 10 neighbors
RNN	Delay	Input delays associated with the recurring connection of the layer

### Metrics

Well-known machine learning metrics were applied to compare the performance of the algorithms. These metrics are based on the confusion matrix, where true positives (TP) correspond to the values classified as positive that were actually positive, and the true negatives (TN) to the values classified as negative that were actually negative. P is the number of records considered to be of the positive class (eyes closed) and N is the number of records of the class considered to be negative (eyes open).:

- Accuracy (ACC): Percentage of cases where the model was correct. A full success rate would correspond to a value of 1.
  15 $$ACC = \frac{TP + TN}{{P + N}}$$
- Sensitivity (SEN): the probability of a positive test, conditioned on being truly positive (true positive rate). A value of 1 corresponds to a full hit rate of positive cases.
  16 $$SEN = \frac{TP}{P}$$
- Specificity (SPE): the probability of a negative test, conditioned on being truly negative (true negative rate). As with the previous metric, a value of 1 in the specification corresponds to all the values considered negative being correctly classified.
  17 $$SPE = \frac{TN}{N}$$
- Matthew correlation coefficient (MCC): correlation coefficient for binary classifications. This metric is represented between the range − 1 and + 1, with 1 representing a perfect prediction, 0 being a random representation, and − 1 total disagreement between the prediction and the observation.
  18 $$MCC = \frac{TP*TN - FP*FN}{{\sqrt {\left( {TP + FP} \right)\left( {TP + FN} \right)\left( {TN + FP} \right)\left( {TN + FN} \right)} }}$$

## Results

The temporal variation of the EEG recordings in BCI applications suggests that classification algorithms that take their historical evolution into account (such as LSTM) would give better results. This section shows the feasibility of using LSTM in BCI systems with a low-cost, wireless EEG device (Emotiv EPOC+).

**Fig. 8:**
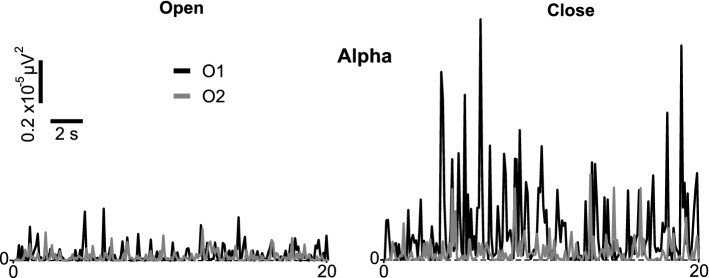
Example of the temporal variation of the power of the alpha band in positions O1 and O2 when the eyes are open or closed

EEG recordings of 13 volunteers were taken using the protocols defined in the previous section. After pre-processing, the time evolution of the alpha-band power is computed using the wavelet transform. Figure 8 shows that the magnitude of the power of the alpha band in the visual area (sensors O1 and O2) is not constant over time and has larger values in the EC state than in the EO state, although, the EC state also has low values that could be confused with the EO state if the power is analyzed with a small time window.

A good BCI must react ideally to the user's action (command) instantly, or at least in the shortest time possible. If the system takes too long to react, the user could run into trouble. In fact, if the BCI’s response is not quick and accurate, a person with reduced mobility could spend considerable time and effort defining a simple action. Consequently, a long journey will take time and be exhausting.

Thus, to deal with these situations and have a more efficient BCI using an LSTM, it is important to define the shortest time window that generates optimal control of the system. Different time windows were used to create the datasets for the LSTM and find the best one.

The time evolution of the alpha-band power recorded in the O1 and O2 positions was used as a dataset to train and validate the classical algorithms and the LSTM network. The dataset was randomly divided to train (75%) and validate (25%) the algorithms. Four metrics, *m*, were calculated: accuracy (ACC), sensitivity (SEN), specificity (SPE) and Matthew correlation coefficient (MCC). The process of randomly dividing the dataset to train and validate the algorithms was repeated 20 times for each subject. Then, the mean value of the metric for each subject *m*_*i*_ is the average of the 20 metrics calculated, *m*_*ij*_, where *i* indicates the subject and *j* identifies the iteration. Finally, the mean and standard deviation using the "*n* − *1*" method (sd.s) of the set of subjects is calculated as

19 $$m = \frac{{\mathop \sum \nolimits_{i = 1}^{I} \left( {\frac{{\mathop \sum \nolimits_{j = 1}^{J} m_{ij} }}{J}} \right)}}{I} = \frac{{\mathop \sum \nolimits_{i = 1}^{I} m_{i} }}{I},$$

20 $$\sigma = \sqrt {\frac{{\mathop \sum \nolimits_{i = 1}^{I} \left( {\frac{{\mathop \sum \nolimits_{j = 1}^{J} m_{ij} }}{J} - m} \right)^{2} }}{I - 1}}$$

where *i* = *1,…,I*, with *I* = 13 and *j* = 1,…,*J*, with *J* = 20. Table 2 shows the mean result of the classical algorithms and Table 3 shows the means for the LSTM network with different time windows. In Table 3, we can see that the accuracy is between 0.7661 and 0.9214, while in Table 2, the accuracy of the classical algorithms is between 0.4854 and 0.6872.

**Table 2:** Metrics of the classic models commonly applied

Algorithm	Accuracy	Sensitivity	Specificity	MCC*
*DTL*				
Fine	0.6032	0.5647	0.6416	0.2088
Medium	0.6225	0.5515	0.6935	0.2530
Coarse	0.6273	0.5244	0.7302	0.2630
*DA*				
Linear	0.6190	0.4334	0.8046	0.2661
Quadratic	0.5989	0.4882	0.7094	0.2228
*SVM*				
Linear	0.6138	0.4808	0.7468	0.2489
Quadratic	0.4854	0.5879	0.3828	− 0.0571
Cubic	0.4896	0.7165	0.2628	− 0.0555
Fine Gaussian	0.6252	0.4274	0.8229	0.2774
Medium Gaussian	0.5968	0.3084	0.8853	0.2419
Coarse Gaussian	0.5135	0.1388	0.8882	0.0842
*KNN*				
Fine	0.5753	0.5734	0.5772	0.1510
Medium	0.6122	0.6492	0.5751	0.2259
Coarse	0.6340	0.5584	0.7097	0.2760
Cubic	0.6104	0.6482	0.5726	0.2224
Weighted	0.5929	0.5776	0.6082	0.1865
Cosine	0.6024	0.6442	0.5607	0.2065
*RNN*				
Delay of 1 s	0.6872	0.6304	0.7439	0.4022
Delay of 10 s	0.6367	0.4497	0.8238	0.2820

The mean is calculated as the average of 13 subjects with 20 runs each

*MCC, Matthew correlation coefficient

**Table 3:** Metrics of the LSTM classifier with different time windows

	Time window (s)									
	1	2	3	4	5	6	7	8	9	10
*Accuracy*										
Mean	0.7661	0.8084	0.8401	0.8735	0.8787	0.8804	0.9094	0.9127	0.9065	0.9214
sd.s	0.1606	0.1496	0.1454	0.1367	0.1199	0.1310	0.1057	0.0911	0.1062	0.0935
*Sensitivity*										
Mean	0.7075	0.7642	0.8139	0.8548	0.8563	0.8558	0.8990	0.8891	0.8967	0.9162
sd.s	0.1948	0.1971	0.1502	0.1721	0.1651	0.1741	0.1217	0.1147	0.1062	0.1267
*Specificity*										
Mean	0.8247	0.8526	0.8662	0.8922	0.9011	0.9050	0.9199	0.9363	0.9163	0.9267
sd.s	0.1616	0.1518	0.1703	0.1153	0.1041	0.1268	0.1097	0.0802	0.1419	0.0640
*Matthew correlation coefficient*										
Mean	0.5397	0.6316	0.6952	0.7563	0.7700	0.7776	0.8276	0.8373	0.8281	0.8534
sd.s	0.3268	0.2907	0.2815	0.2662	0.2279	0.2429	0.2014	0.1687	0.1939	0.1769

The sample mean and standard deviation are calculated as the average of 13 subjects with 20 runs each

The Friedman test yielded a statistically significant difference in the accuracy depending on the time windows, with a test statistic value F = 20.462 > χ^2^(2) = 7.815 and a significance level *p*-value = 0.000036. Then, to locate time windows with significant differences, post hoc studies with Wilcoxon signed-rank test were applied between different windows. The *p*-values obtained from these comparisons are shown in Table 4. *p*-values greater than 0.05 mean that there is no significant improvement to the LSTM algorithm when comparing the classifier accuracies using two different time windows. Therefore, as this table shows, there is no significant improvement for window sizes longer than 7 s.

**Table 4:** *p*-values of the Wilcoxon signed-rank test to compare the accuracy of LSTM algorithms with different time windows

	Accuracy—Wilcoxon rank-sum test: *p*-values								
	Time windows (s)								
	2	3	4	5	6	7	8	9	10
Time windows (s)									
1	***	***	***	***	***	***	***	***	***
2		***	***	***	***	***	***	***	***
3			***	*	***	***	***	*	***
4				0.5878	0.5759	*	*	0.0590	*
5					1	*	*	0.0573	*
6						*	*	*	*
7							0.8445	0.8925	0.3395
8								0.8393	0.3757
9									0.1464

*p < 0.05; ***p < 0.001

**Fig. 9:**
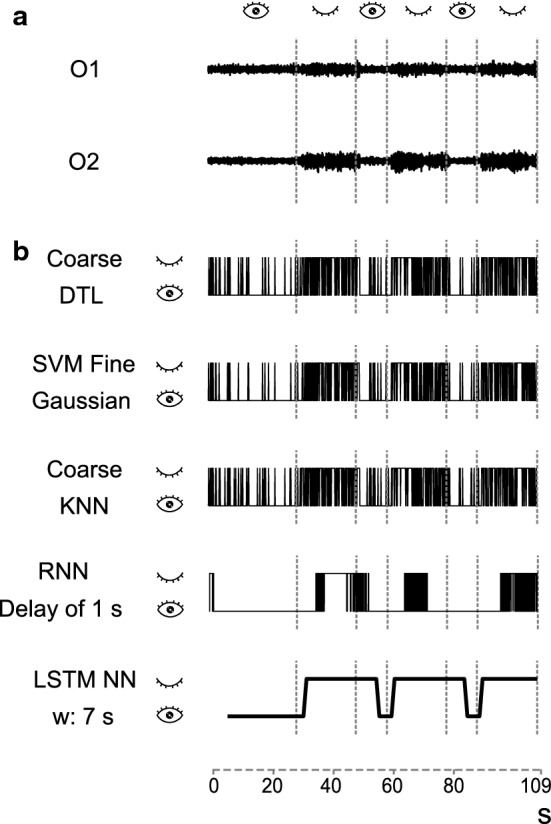
Classification results with different algorithms obtained for one user. **A** Power in the alpha band recorded with eyes open and closed in O1 and O2 position. Eyes are closed for 20 s. **B** Classification results for the DTL, SVM, KNN and RNN algorithms with the configurations that yielded the best training accuracy (Table 1) and an LSTM classifier with a time window of 7 s

Once the classifier algorithms were trained and validated, they were tested with new records. The paradigm used for the test recordings consisted of three EC states, each of which is t seconds long, with an EO state in between. Figure 9 shows the classification output for a user with different algorithms. The signals at the top (Fig. 9a) are EEG power records from the O1 and O2 sensors. Figure 9b shows the results using the best classifiers according to Table 2, and an LSTM classifier with a time window of 7 s.

**Fig. 10:**
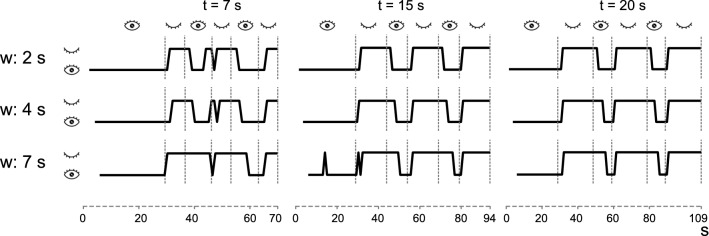
Classification results for LSTM with time windows of 2, 4 and 7 s for eyes closed time periods of 7, 15 and 20 s

Figure 10 represents the classification of the same recordings as in Fig. 9 with the LSTM and different time windows (w): 2, 4 and 7 s. Also, long EC states (t) were defined with different durations: 7, 15 and 20 s.

**Fig. 11:**
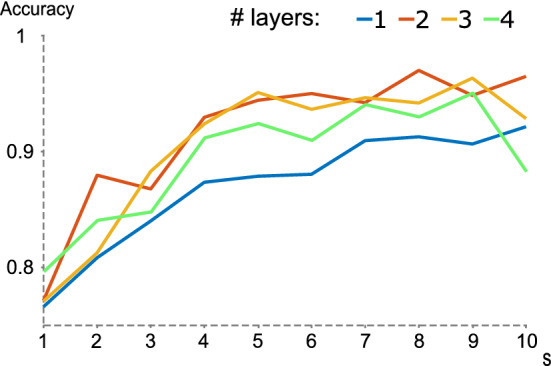
Representation of the different accuracies (y-axis) obtained with various neural networks having different numbers of LSTM layers (each layer defines 8 neurons). Windows between 1 and 10 s have been applied (x-axis)

In addition, to test the network architecture, the number of LSTM layers has been increased (using eight neurons in each one) and their accuracy has been evaluated, yielding the result shown in Fig. 11 with the different time windows used. Note the elbow that is formed in the time windows between 5 and 7 s. As for the results, when applying several layers (2, 3 and 4), the evolution of the accuracy is similar, while using a single layer has lower values.

## Discussion

In this study, we have chosen a low-cost wireless EEG device, namely the Emotiv EPOC + , because it is wireless and easy to fit, which facilitates its use in BCI applications, for example in situations where people with reduced mobility wish to interact with their environment through computers. In addition, because it is a low-cost device, it can be used by a greater number of potential customers who will have less purchasing power. However, a low-cost wireless EEG device has a number of disadvantages compared to clinical devices in terms of the quality of the acquired signal. Therefore, in this study, we looked at the possibility of applying an LSTM network to the EEG recorded by a low-cost wireless EEG device and the need to choose the appropriate time window to improve accuracy. We also wanted to show that in this case, an LSTM network can yield better results than classical classification algorithms.

In order to study and compare the selected classification algorithms, the brain activity of 13 volunteers was measured while keeping their eyes closed or open, depending on the external stimuli received. Then, the purpose of the classifier is to decide whether the subject has their eyes closed or open. As was shown at the beginning of the results section, there is a clear contrast in brain signals when a user is in the EO or EC state, which is present in the alpha-band power signal, Fig. 8. The results obtained by Gopika Gopan et al. (2017) are similar, demonstrating a clear difference between these two states with an accuracy of 77.92%. But when comparing the classic classification algorithms, Table 2, with the LSTM networks, Table 3, we see that the latter are more accurate, yielding an accuracy that is at least 8% higher than standard algorithms. Although, Fig. 8 shows a clear difference between EEG recordings in the eyes open and eyes closed states, if we analyze shorter time intervals, such as one second or less, the difference is not so obvious. Since the power does not remain constant, a low power may be measured in both the EO and EC states. In this case, traditional classification algorithms cannot discern the difference between the two states. Therefore, when the classification algorithms are trained directly with the values of the alpha-band power signal that change with time, their accuracy is worse and the records must be processed to extract other characteristics that can improve the classification over a longer time window. When we focus on the sensitivity, specificity and MCC of these classic algorithms, Table 2, we see this behavior reflected: the accuracy average is 59.71% and the sensitivity (ratio of correct positives and all the elements classified as positive) and specificity (negative elements in relation to all elements classified as negative) are 52.38% and 67.04%, respectively. But in the case of MCC, we see that most of these algorithms have a value very close to 0, which means that the classification of these algorithms is almost random. Furthermore, it is observed that the application of the RNN, which works with previous information of the data, does not depart from the accuracies obtained with the rest of the algorithms. However, a classifier such as the LSTM, which takes into account the information in the previous time instants, produces better results. Table 3 shows that the accuracy (76.61–92.14%), sensitivity (70.75–91.62%) and specificity (82.47–93.63%) of LSTM networks are higher than the classical algorithms. In addition, the MCC is distributed between the range of 0.53 and 0.85, which means that its prediction is quite good (a perfect prediction is when MCC is equal to 1).

An important factor in properly training an LSTM network is choosing the duration (time window) of the records that are passed to the network. Other author, like Kumar et al. (2019), used a single time window that was 25% of the trial duration and with an overlap of approximately 5%. However, in this paper, the time window size is changed to compare its relationship with the algorithm’s performance.

The results show that LSTM classifiers generate a better accuracy with longer windows—from a mean accuracy of 76.61% with a 1-s window size to 90.94% with 7 s. From this point on, the accuracy values are around 91%. In our case, no pre-selection of the signal was done when applying the proposed methodology. Other authors do select the best segments to build their dataset, which is not applicable in real-time systems; for example, Piątek et al. (2018) obtained an accuracy of over 96% with different classification algorithms with a time window of 10 s.

From the results presented in Tables 3 and 4, we concluded that after 7 s there is no significant improvement in the classification.

Finally, EEG records presenting a more realistic situation with EO and EC states were used to test how the different classifiers will work in a real BCI (Fig. 9a). Figure 9b shows the outcomes of the Coarse DTL, SVM Fine Gaussian, Coarse KNN algorithms and RNN with a delay of 1 s, which had the best accuracy in Table 2. Although the user is in a fixed state (EC or EO), the responses of these four classifiers change continuously between EC and EO. In contrast, the LSTM results are more stable and closer to the user’s behavior. It should be noted that the RNN applies a dynamic temporal behavior as well, but does not achieve the constancy of actions present in the LSTM.

However, there is one aspect to highlight in Fig. 10, where different lengths of the EC state (t) are used in the protocol and the applied LSTM uses different time windows (w). Although the optimal result obtained in Table 3 is 7 s, we see how lengthening the window size affects the EO transition by adding a substantial delay, which could decrease the accuracy. It is necessary that the sequences to be classified in the LSTM be formed by exclusive characteristics of each state in order to be correctly classified. If they are formed by several classes, delays would be created because until the new state is predominant, the result of the classification will not change. That means that in a real system, it is necessary to establish a compromise between accuracy and response times since an LSTM-based BCI user has to know the duration of the time window over which to give his commands so they can be detected.

It should be noted that the network used and applied in the real context has a single LSTM layer which obtains higher accuracy than the classical algorithms. The results can be improved by adding more layers, as seen in Fig. 11.

## Conclusions

In general, the algorithms applied can be considered, on the one hand, as classical algorithms, such as ML algorithms and, on the other hand, as DL algorithms, such as LSTM. In both cases, the quality and reliability of the data are essential to ensure efficient performance, even in the case involved in this research. In general, DL techniques usually require a large dataset and have a higher computational cost due to the complexity of the operations they perform. In this case study, the training was not significantly different, and the results were comparable, with the LSTM results standing out thanks to the sequential processing performed by this layer.

In a BCI context, although there is an extensive literature on EEG-based BCI with LSTM networks, there are not enough studies focused on the time window parameter, which is essential for its success. Our results highlight different aspects that we must take into account to implement an LSTM network in a BCI.

In our case study, the task of distinguishing the EO and EC states is complicated due to the changing evolution of the EEG signal. Most of the peaks in the alpha-band power in the EO state are low compared to those in the EC state. However, in an EC state, some values of power may be similar to those in the EO state. Therefore, this fact makes the classifier require extra information to differentiate the two states, since the discrete values of power do not have enough information to differentiate the states correctly.

There are different approaches to solve this problem; for example, using a threshold dependent on the maximum value of the peak powers detected in each state. When several consecutive power peaks of the EEG signal are detected and exceed this threshold in a predefined period, it is considered to be in the EC state, and if not, in the EO state. However, this method involves making several assumptions such as: assuming the behavior of the power, determining how often the power peaks appear, defining a threshold, etc. Therefore, a detailed study of each user’s EEG is necessary. In this work, we demonstrate how an LSTM neural network can elegantly solve this problem.

The outcomes obtained with the LSTM have demonstrated that it is able to obtain a temporal pattern in the EEG signals, as other authors have demonstrated (Li et al. 2016; Wang et al. 2018). Based on our results, the importance of the time window in an LSTM is vital because there is a relationship between the time window sequence used and the accuracy of the classifier. In addition, the LSTM models proposed yielded better outcomes than the classical algorithms, which would need a longer pre-processing of the data to obtain the same level of performance. On the other hand, unlike an RNN network, LSTM can store information over long periods of time, improving the classification results. In summary, there is a relationship between the time window and the accuracy of the LSTM classifier which determines the efficacy and accuracy of a BCI system.

And finally, as a generic conclusion, despite the good results obtained with the EEG, there are other methods, such as EMG (Usman et al. 2021) or video eye tracking (Ibrahim et al. 2021) that could obtain better results in the proposed task. But we must not forget the potential of the system proposed here, which can be applied to other tasks such as providing feedback to aid with motor diseases (Miao et al. 2020; Pimenta et al. 2021), neuromarketing (Gers et al. 2000) or the classification of more elaborate brain tasks (Pfurtscheller et al. 1998; Kumar et al. 2019; Netzer et al. 2020) (to which the same study conducted in this work can be applied).

## Data Availability

The data obtained for this manuscript is available upon request to the corresponding author.
